# Detectability of landscape effects on recolonization increases with regional population density

**DOI:** 10.1002/ece3.1527

**Published:** 2015-06-18

**Authors:** Anna-Sara Liman, Peter Dalin, Christer Björkman

**Affiliations:** Department of Ecology, Swedish University of Agricultural SciencesUppsala, Sweden

**Keywords:** Disturbance, population dynamics, regional synchrony, scale, time series, willow short rotation coppice

## Abstract

Variation in population size over time can influence our ability to identify landscape-moderated differences in community assembly. To date, however, most studies at the landscape scale only cover snapshots in time, thereby overlooking the temporal dynamics of populations and communities. In this paper, we present data that illustrate how temporal variation in population density at a regional scale can influence landscape-moderated variation in recolonization and population buildup in disturbed habitat patches. Four common insect species, two omnivores and two herbivores, were monitored over 8 years in 10 willow short-rotation coppice bio-energy stands with a four-year disturbance regime (coppice cycle). The population densities in these regularly disturbed stands were compared to densities in 17 undisturbed natural *Salix cinerea* (grey willow) stands in the same region. A time series approach was used, utilizing the natural variation between years to statistically model recolonization as a function of landscape composition under two different levels of regional density. Landscape composition, i.e. relative amount of forest vs. open agricultural habitats, largely determined the density of re-colonizing populations following willow coppicing in three of the four species. However, the impact of landscape composition was not detectable in years with low regional density. Our results illustrate that landscape-moderated recolonization can change over time and that considering the temporal dynamics of populations may be crucial when designing and evaluating studies at landscape level.

## Introduction

The spatial and temporal scale at which ecological studies are performed can greatly influence our understanding of the composition of natural communities and their dynamics (Wiens 1989; Levin 1992; Chase and Leibold 2002; Hastings 2004, 2010; Hortal et al. 2010; Wang and Loreau 2014). The time scale of a process increases with the spatial scale at which it is operating, i.e. broad-scale processes operate on longer time scales (Wiens 1989). The temporal extent of a study will, therefore, limit the patterns and processes that can be discovered (Wiens 1989; Hastings 2010). Hierarchy theory predicts that processes operating on finer spatial scales can be constrained by those that influence the system on broader spatial scales; e.g., variation between landscapes can be averaged out as climate and topography becomes increasingly important (Allen and Starr 1982; Sutcliffe et al. 1996). Due to such hierarchical effects, the temporal scale of a study must be adjusted to capture also large-scale variation, or there is a risk that fine-scale patterns are wrongly estimated.

Our current understanding of how population and community processes relate to landscape patterns relies to a large extent on studies of disturbance–recolonization events (Turner 2010). The degree of recolonization following a local extinction reflects the population dynamics in surrounding more stable patches in the landscape (Tscharntke et al. 2012). However, the probability of identifying variation in these fine-scale processes can be affected by regionally synchronized population growth. For example, extreme weather events can push population densities in all landscapes below thresholds where density-dependent dispersal is limited, which will reduce the detectability of landscape-moderated recolonization. Most studies in landscape ecology to date only cover snapshots in time, despite the risk of identifying false patterns by overlooking the dynamics of populations and communities (Chaplin-Kramer et al. 2011). The few studies that do utilize repeated measures in time, e.g., Menalled et al. (2003) and Chaplin-Kramer et al. (2013) conclude that the temporal extent of the study was critical for the results.

In this study, we used a longer time series and utilized the natural variation between years, to explore the relationship between landscape composition and population density during patch recolonization at different regional density levels. As a model system, we used willow short-rotation coppice (SRC) bio-energy stands with a four-year coppice cycle and compared these to undisturbed natural willow stands in the same region. Populations of four interacting insect species were monitored over 8 years: two leaf feeding willow beetles *Phratora vulgatissima* L. and *Galerucella lineola* F. (Coleoptera: Chrysomelidae) and two of their main predators, the omnivorous bugs *Orthotylus marginalis* Reut. and *Closterotomus fulvomaculatus* De Geer (Heteroptera: Miridae).

Our aim with this study was to explore the implications of temporal variation in population density synchronized at a regional scale, in order to understand the importance of patch context for recolonization and community assembly. We hypothesized that regionally high population densities increase the – detectable – impact of landscape composition on insect recolonization of disturbed habitat patches.

We predicted that: 1. Recolonization and population growth among all interacting species following a coppicing disturbance event should be landscape-moderated when regional densities are high. Population densities of both species of omnivorous mirids should be higher in landscapes with a high proportion of open habitat, while densities of both species of willow leaf beetles should be higher in more forest-dominated landscapes, i.e., with a lower proportion of open habitat. This pattern was expected because population densities of omnivorous mirids are higher and more stable over time in natural grey willow stands growing in nitrogen-rich environments, i.e., open agriculture-dominated landscapes (A-S. Liman et al. unpubl. data). Willow leaf beetle densities are lower in natural grey willow stands in open habitats partly due to high predation pressure from omnivorous mirids (Dalin 2006). 2. When population densities, for whatever reason, are regionally low, there will be no difference in population densities of mirids and willow leaf beetles between landscapes with different proportions of open habitat. This is because density-dependent dispersal should be low in all landscape types when regional population density is low.

To our knowledge, this is the only study at landscape scale to date that utilizes a time series approach in order to explore how the relationship between patch context and population density varies at different levels of regional density.

## Materials and Methods

### Study system

We used two different willow (Salicaceae) systems: managed SRC willow (*Salix viminalis* L.) and natural grey willow (*Salix cinerea* L.). Willows in SRC forestry are grown on arable land, mainly for biomass production, and the predominant species used is *S. viminalis*.

Leaf beetle outbreaks in willow SRC can cause significant losses of biomass and even shoot death (Björkman et al. 2000; Bell et al. 2006). Omnivorous mirids predate on leaf beetle eggs and larvae and can prevent outbreaks in both managed and natural willow systems (Björkman et al. 2004; Dalin 2006). The omnivorous mirid bugs hibernate in the willow stand, as eggs buried in the stems (Björkman et al. 2003). When all stems in a stand are cut back, all mirid eggs are removed. The willow leaf beetles hibernate off-site and recolonize the re-sprouting willows again in spring, which make them less sensitive to the direct effects of winter coppicing (Sage et al. 1999; Björkman and Eklund 2006). Severely reduced local populations of omnivorous mirid bugs allow for high leaf beetle population growth rates in re-sprouting willows the following spring (Björkman et al. 2004). Landscape level site selection could be used to facilitate recolonization of omnivorous mirid predators and limit the risk of willow leaf beetle outbreaks.

The grey willow is unmanaged and form dense stands along small streams, ditches and pastures and at forest edge. Grey willow stands are common in the same landscapes as SRC willows and were together with *Salix caprea* L. and free living *S. viminalis* identified as the main source for leaf beetle recolonization of willow SRC in the UK and Ireland (Sage and Tucker 1998). *Salix viminalis* and *S. cinerea* are chemically similar and host very similar insect communities (Volf et al. 2015). Population densities of the studied mirid and leaf beetle species vary within similar ranges in the two *Salix* systems (Dalin et al. 2009).

### Study sites

The study area covers about 50 × 75 km with a total of 38% open land cover and 54% forest land cover. We selected 10 SRC willow stands and 17 grey willow stands from a database of 32 managed and 17 natural stands, monitored 1999–2014. The grey willow stands were located along a gradient from forest edge to more open habitats. These natural willow stands could not be geographically paired with the SRC willow stands. Therefore, we decided to use the grey willow data only as indicators of regional population densities.

The selected SRC stands were of approximately the same age (established 1990–1994), and coppiced for the third and fourth time in January–February 2003 and 2007. By selecting stands in the same coppice cycle, we could compare recolonization and population buildup during the same years and under the same weather conditions. Repeating the analysis on a different set of stands could have given an indication of the robustness of the results. Unfortunately, there were not enough stands in coppicing cycles with a 4-year offset from the ones analyzed (e.g., 1999–2002, 2003–2006).

### Population data

Abundances of leaf beetles (*Phratora vulgatissima*, *Galerucella lineola*) and their predators (*Orthotylus marginalis*, *Closterotomus fulvomaculatus*) in the selected willow stands were estimated annually in early June over 8 years (2003–2010). Population densities were estimated using a “knockdown” sampling method (Björkman et al. 2004; Dalin 2006). All insects on current years shoot were dislodged into a white plastic container; individuals of the four focal species were counted and released back to the shoot. The number of samples differed between stands but was proportional to stand size (Pearson *r* = 0.61).

### Geographic data, landscape composition

ArcGIS 10.0 (ESRI 2010) was used to plot and analyze geographic data. Explanatory variables estimated from the geographic data were SRC willow patch area, the total area of SRC willow and the proportion of open and forest land cover in the landscapes surrounding the SRC willow stands. The stands were delineated using aerial photography and spatially explicit data on agricultural land use (minimum unit area 0.01 ha) from the Integrated Administration and Control System (IACS) provided by the Swedish Board of Agriculture. The spatial land cover data were sourced from the Swedish mapping, cadastral, and land registration authority. Landscape composition was extracted from the GSD-Topographic map, scale 1:50,000. Closed land cover polygons were broadly reclassified as open land cover, forest, and other land cover.

Landscape composition was analyzed at three spatial scales represented by buffer zones from the stand edge at 500 m, 1000 m, and 1500 m. Buffer zones were adjusted according to change in stand perimeter, thus always representing the same distance from stand edge. As a result, buffer zone size varied between years but was always proportional to stand size. The proportion of open land cover and forest land cover was highly correlated across all scales (500 m *r* = −0.95, df = 8, *P* < 0.001; 1000 m *r* = −0.90, df = 8, *P* < 0.001; and 1500 m *r* = −0.90, df = 8, *P* < 0.001). Thus, we choose to include open land cover and exclude forest cover from the analyses as a positive relationship with increasing open habitat cover would essentially mean the same as a negative relationship with the proportion of forest cover.

The proportion of open land cover was also highly correlated across scales (500 m vs. 1000 m *r* = 0.96, df = 8, *P* < 0.001 and 500 m vs. 1500 m *r* = 0.92, df = 8, *P* < 0.001), which implies that landscapes were very similar at scales below 1500 m and that variation among landscapes occurred at distances greater than 1500 m. Dispersal distances have a strong influence on the spatial extent at which landscape structure best predicts abundance of the species and can therefore be used to determine the most appropriate landscape scale (Jackson and Fahrig 2012). In this system, the 500-m scale was determined to be most adequate, as it captures twice the post-hibernation dispersal distance of leaf beetles and recorded maximum dispersal distances for mirids with similar life histories (Waloff and Bakker 1963; Kendall and Wiltshire 1998; Sage et al. 1999). The area of the buffer zones at the 500-m scale had a mean of 141 ha (range = 101–196 ha) in 2003 and 139 ha (range = 101–190 ha) in 2007. None of the 500-m scale buffer zones overlapped.

### Land-use change

Land-use management (e.g., fertilization) and crop type in the matrix between stands can affect recolonization after coppicing. Therefore, we validated our results with respect to observed trends in land-use change at the landscape scale and the regional scale. At the landscape scale, we estimated the annual proportion of SRC willow in the landscape surrounding all stands (based on a 1500-m buffer from each site edge). At the regional level (the 55 × 75 km study area), we estimated trends in arable land-use over time. Arable land use was categorized as SRC willow, other perennial crops (including fallow land), or annual crops.

### Statistics

**Figure 1:**
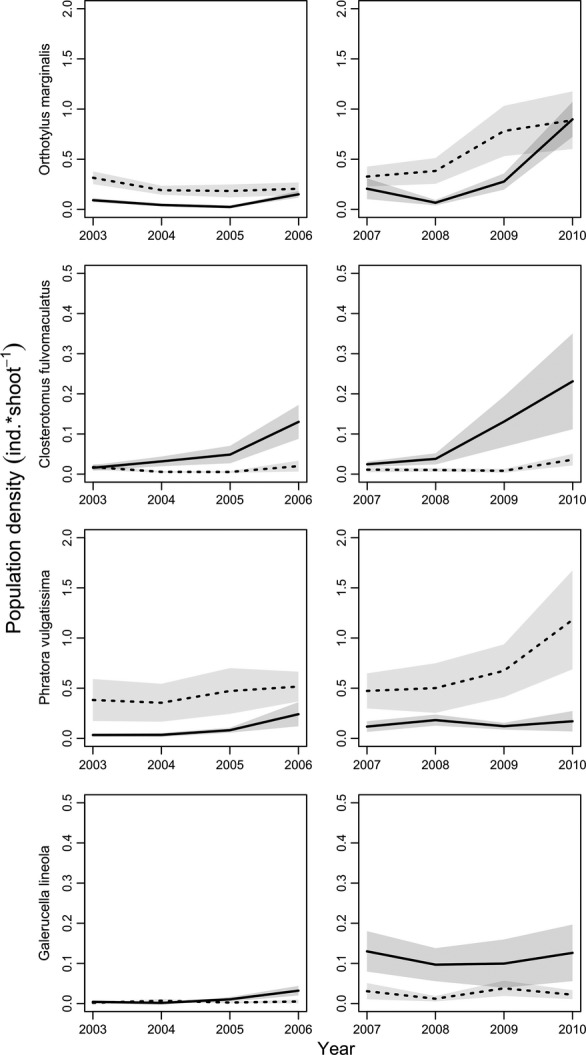
Population densities (ind*shoot^−1^) in 17 natural grey willow stands (dotted lines) and 10 SRC willow stands (solid lines) in two coppice periods, 2003–2006 and 2007–2010 of two mirid predators (*Orthotylus marginalis*, *Closterotomus fulvomaculatus* and leaf beetle herbivores (*Phratora vulgatissima* and *Galerucella lineola*). Densities are presented as mean values with standard errors. Note the difference in scales on the *y*-axes.

The relationship between population density and proportion of open habitat in the surrounding landscape was estimated using generalized linear mixed models (GLMMs) and count data (Poisson distribution, log link) with an offset for log (number of samples) in each stand. Different levels of population densities of all species in the two coppicing periods motivated modeling each period separately, i.e., a total of eight models (Fig.1). This two model approach allowed us to compare the relationship at two different levels of regional population density.

Fixed effects in the initial model were the proportion of open habitat within 500 m of the stand edge, the proportion of SRC willow in the surrounding landscape, patch area, and observation year. Stand was treated as a random effect, to account for potential spatial autocorrelation between stands. Temporal autocorrelation was accounted for by introducing a first-order autoregressive structure, with observation year nested within stand.

The variable proportion of SRC willow in the landscape was not significant in any of the models and was therefore excluded from the analysis. Patch area was negatively correlated to the proportion of open land cover (Pearson *r* = −0.7, *P* = 0.02), meaning that patch area was generally smaller in more open landscapes. To determine the relative size of the landscape variable versus the patch area variable, we compared two types of models only differing in explanatory variables (open habitat and year or patch area and year). We found that parameter estimates for patch area were generally close to zero and of the same sign as the open habitat estimate (Appendix S1 Fig. S1). However, the negative correlation between the two explanatory variables implies that they had opposite effects on population density. The patch area variable was, therefore, removed from the final models.

Over-dispersion, mainly caused by zero observations, was corrected using a quasi-likelihood model, where the dispersion parameter is estimated from the data (Zuur et al. 2009). An alternative to quasi-likelihood models was using zero-inflated mixed models. However, we found no function that could fit a zero-inflated mixed model with an autocorrelation structure. The second best alternative was therefore a complementary GLMM analysis split in two parts: (1) using only counts ≥1, which allowed us to determine whether the zero observations in the data influenced the overall results, and (2) using a binomial distribution to diagnose the probability of zero observations (Appendix S2 Fig. S2).

Analyses were performed in R 2.14.0 (R Development Core Team. 2014) using the MASS package glmmPQL function for GLMMs (Venables and Ripley 2002).

## Results

### Recolonization and population density

Regional mean densities of *O. marginalis* and *G. lineola* in both SRC and in natural willow stands were lower in 2003–2006 than in 2007–2010 (Fig.1). In the natural willow stands, densities of *P. vulgatissima* were lower in 2003–2006 than in 2007–2010, but mean densities were similar in the SRC willow stands in the two coppice periods (Fig.1). In the SRC willow stands, mean densities of *C. fulvomaculatus* were lower in 2003–2006 than in 2007–2010, but mean densities were similar in the grey willow stands (Fig.1).

**Figure 2:**
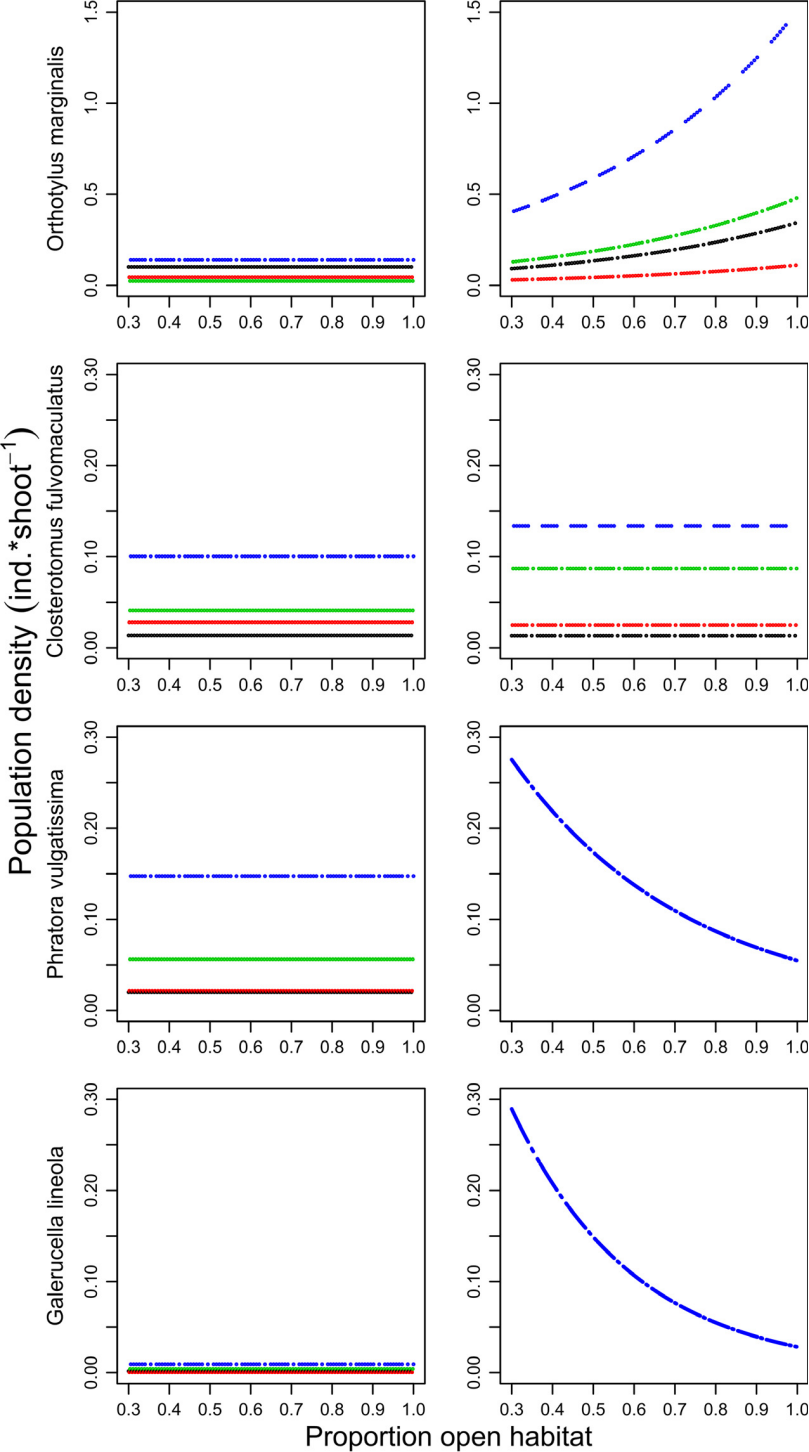
Model predicted population densities (ind*shoot^−1^) as a function of the proportion of open habitat in the surrounding landscape and/or year after harvest, two 4-year periods after coppice harvests of two mirid predators (*Orthotylus marginalis*, *Closterotomus fulvomaculatus* and two leaf beetle herbivores (*Phratora vulgatissima* and *Galerucella lineola*) in SRC willow stands (*N* = 10). Predictions in the left column are based on data from 2003 to 2006 and in the right column on data from 2007 to 2010. Note that only variables with an overall significant effect on population density were used for the model predictions (single dotted line = no difference between years, four dotted lines = difference between observation years (red = year 1, black = year 2, green = year 3, blue = year 4).

Landscape composition had an effect on densities of re-colonizing populations following willow coppicing in three of the four studied species (Fig.2). Population densities of the most common mirid, *O. marginalis,* were higher in stands surrounded by landscapes with higher proportion open agricultural fields (Fig.2). Recolonization by the less common mirid predator *C. fulvomaculatus* showed no response to landscape composition. Population densities of both the two willow leaf beetles decreased along the same gradient (Fig.2). These patterns were, however, only found in high population density years (2007–2010) (Fig.2, Appendix S1 Fig. S1). The impact of landscape composition on recolonization could not be detected in low density years (2003–2006) in any of the species (Fig.2, Table1 and Appendix S1 Fig. S1).

**Table 1:** Analysis of variance for GLMMs (Poisson distribution and log link) describing population density as a function of proportion open habitat and year in four insect species. The models were fitted using quasi-likelihoods, with stand as a random effect and a first-order autocorrelation structure to account for spatial and temporal dependence between observations

Year	Species	Fixed effect	Estimate	SE	df	Chisq	*P*-value
2003–2006	*O. marginalis*	Open habitat	0.88	0.93	1	0.81	0.37
		Year	−3.39	0.70	3	25.07	<0.001***
	*C. fulvomaculatus*	Open habitat	−0.34	1.54	1	0.13	0.72
		Year	−3.06	1.09	3	30.62	<0.001***
	*P. vulgatissima*	Open habitat	−1.31	1.72	1	0.53	0.46
		Year	−2.21	1.23	3	51.13	<0.001***
	*G. lineola*	Open habitat	−2.80	1.96	1	3.64	0.06
		Year	−3.02	2.00	3	63.64	<0.001***
2007–2010	*O. marginalis*	Open habitat	1.92	0.77	1	6.81	0.009**
		Year	−2.87	0.69	3	29.65	<0.001***
	C. fulvomaculatus	Open habitat	−1.10	1.62	1	0.48	0.49
		Year	−2.43	1.13	3	88.88	<0.001***
	*P. vulgatissima*	Open habitat	−1.85	1.13	1	3.99	0.046*
		Year	−0.91	0.69	3	2.39	0.50
	*G. lineola*	Open habitat	−3.32	1.25	1	6.85	0.009**
		Year	−0.26	0.66	3	1.12	0.77

Estimates and SE are the parameter estimates for the fixed effects and their associated standard errors. Estimates and SE for the year variable are mean values across all levels of the factor.

* *P* ≤ 0.05

** *P* ≤ 0.01

*** *P* ≤ 0.001.

The model predictions for 2007–2010 show an average 270% increase in *O. marginalis* population densities, when increasing the proportion of open habitat in the landscape from 30% to 100% (Fig.2, right panel). In the same years and within the same range of landscape composition, average model predictions indicated a decrease in leaf beetle population density by 78% (*P. vulgatissima*) and 89% (*G. lineola*) (Fig.2, right panel).

The complementary analysis using only observations with abundance ≥1 indicated that zero observations did not influence the overall results, i.e., the general results were unchanged when zeros were excluded (Appendix S2 Fig. S2a). The probability of zero observations did not change with proportion of open habitat in any of the models (Appendix S2 Fig. S2b).

### Land-use change

The total proportion of SRC willow in surrounding landscape did not explain the spatial variation in density of any of the four insect species (eight models). The average proportion of SRC willow land cover in our study landscapes was relatively constant over the period 2003–2010, apart from a slight average decrease (Appendix S3 Fig. S3). Land use at a regional scale (the entire study area) was also fairly similar with respect to proportional coverage of SRC willow, other perennial land uses and annual crops (Appendix S3 Fig. S3).

## Discussion

Recolonization of SRC willow stands after coppicing was related to landscape composition; population densities of the most common mirid species increased, whereas densities of both willow leaf beetles decreased with increasing proportion of open habitat in the surrounding landscape. However, these patterns were only detected in years with high regional population densities. A reasonable explanation for the variation in outcomes over time is the occurrence of a regional-scale factor, e.g., unfavorable weather conditions could have reduced populations below thresholds where density-dependent dispersal from populations in all landscapes becomes limited. This suggests that awareness of variation in population density, due to e.g., regional synchrony, can be highly relevant for understanding landscape-moderated patterns of recolonization.

Ritchie (2000) illustrated how extremes in abiotic conditions can mask the bottom-up control of herbivore abundance in a nitrogen-limited system. The author concluded that interactions between abiotic conditions and local ecological processes can blur spatial variation at patch level. We interpret our results as probably arising from a somewhat similar phenomenon, but extended to a landscape scale pattern. In years when populations are regionally synchronized at low density levels, bottom-up and top-down effects (e.g., host plant nitrogen status and predation pressure) in source habitats explain less of the variation in population density among SRC willow stands.

Despite the apparent problems with ignoring the dynamics of populations, most landscape scale studies use snapshot estimates (Chaplin-Kramer et al. 2011). There are currently very few studies based on repeated measures over several years, taking into account different levels of regional density. In addition, few previous studies have looked simultaneously at temporal dynamics of interacting species at a landscape scale, but see Oliver et al. (2010) and Chaplin-Kramer et al. (2013), or have considered the actual effects of interactions between weather and landscape composition, but see Cormont et al. (2014). Our study illustrates several advantages of considering not only snapshot estimates of single species, but rather longer periods of temporal dynamics of interacting populations, as patch context can have such a variable impact on community composition over time. However, because of the low temporal replication, these results can only be used to demonstrate, rather than explicitly test, how regional density levels can influence the conclusions of a landscape study.

A possible disadvantage with using a time series approach is, as previously mentioned, potential changes in land-use and management regimes over time. Another problem is to determine how many repeated observations are needed to capture “enough” variation. There are alternatives to using time series data, e.g., could snapshot studies be designed to capture different levels of population density by increasing the spatial scale. This could, e.g., be done through comparisons between different geographic areas that experience different abiotic conditions. However, a spatial approach has the disadvantage of increasing the number of confounding factors and thus the variability in the data.

Using two consecutive time series, we have indirectly related variation in recolonization of managed habitats to regional density levels in undisturbed natural habitats, the source of many recolonizing individuals. Our willow–insect model system is likely to be representative of a range of systems, as induced patterns of spatially synchronized population dynamics have been reported for numerous taxa and trophic levels (Liebhold et al. 2004). We have assumed, although not explicitly tested, that synchronous exogenous random factors (e.g., temperature) produce the observed patterns. However, the causes of spatial synchrony are often difficult to disentangle as it may, e.g., also arise from dispersal among populations or be indirectly mediated through trophic interactions (Kendall et al. 2000; Bjørnstad and Bascompte 2002; Liebhold et al. 2004). Our results support the idea of bringing together studies on population dynamics with landscape ecology to gain a better understanding of the spatial dynamics of populations in managed landscapes.

We conclude that the overall importance of the landscape setting for species recolonization and abundance can be wrongly interpreted if the temporal scale of the study is too short. The results presented here support the suggestion that ecologists would benefit from considering the dynamics of populations and communities, e.g., using longer time series of observations in landscape ecology (Tscharntke and Brandl 2004; Chaplin-Kramer et al. 2011).

## Data Accessibility

Population density and land cover data: uploaded as online supporting information.

## Supporting Information

**Appendix S1.** Population density related to patch area. **Appendix S2.** Population density related to proportion open habitat (alternative analysis). **Appendix S3.** Land use dynamics. **Figure S1.** Comparison between patch area and open landscape model parameter estimates. **Figure S2.** Alternative analyses with the aim to diagnose the impact of absence data (zero observations) on the relationship between population density (ind*shoot^−1^) and the proportion of open habitat. **Figure S3.** Land use changes over time at landscape and regional scales.

**Appendix S4.** Population and land cover data. **Data S1.** Population densities – SRC willow stands.

**Data S2.** Population densities – grey willow stands.

**Data S3.** Land cover data.

**Metadata.** Population and land cover data.
