# Supplementary material for: Detectability of landscape effects on recolonization increases with regional population density
Source: Ecol Evol. 2015 Jun 18;5(13):2694–702. doi: 10.1002/ece3.1527 (PMC4523364; doi:10.1002/ece3.1527)
Supplement: Supplementary file 1 [file ece30005-2694-sd1.docx]

**Appendix S1**

**
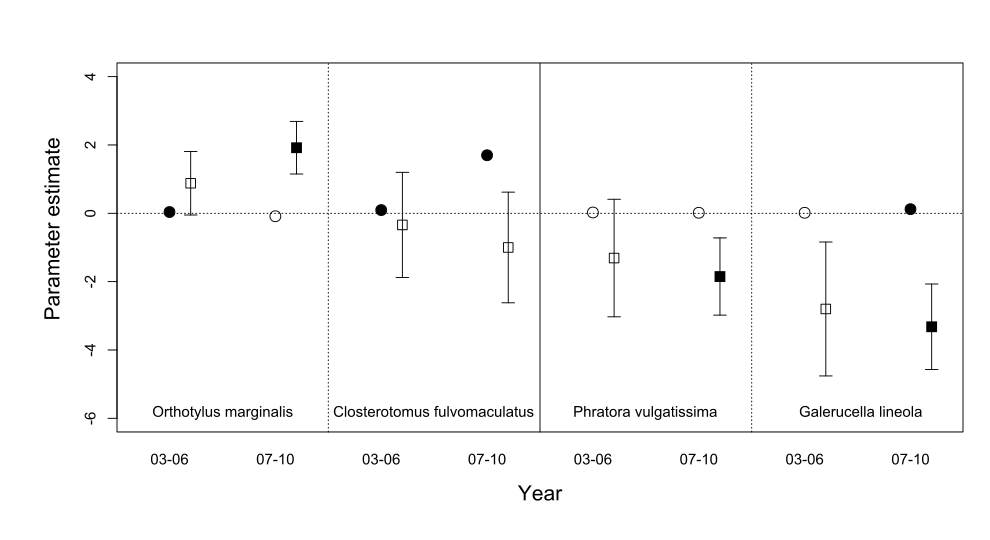
 Figure S1.** Mean parameter estimates in models with data from different years (2003-2006 or 2007-2010) and with two different explanatory variables, i.e. 1) patch area (circles) or 2) proportion of open habitat in the surrounding landscape (squares). The left panel show results for the two mirid predator (*O. marginalis* and *C. fulvomaculatus*) and the right panel show results for the two leaf beetles herbivores (*P. vulgatissima* and *G. lineola*). Closed symbols show significant effects (Table 1). Error bars for open habitat estimates show standard errors. Standard error for patch area mean estimates were all <±0.15 (smaller than the point symbol) and are therefore not visible. Note that patch area was negatively correlated to the proportion of open land cover (Pearson r=-0.7, p=0.02).

**Appendix S2**

**
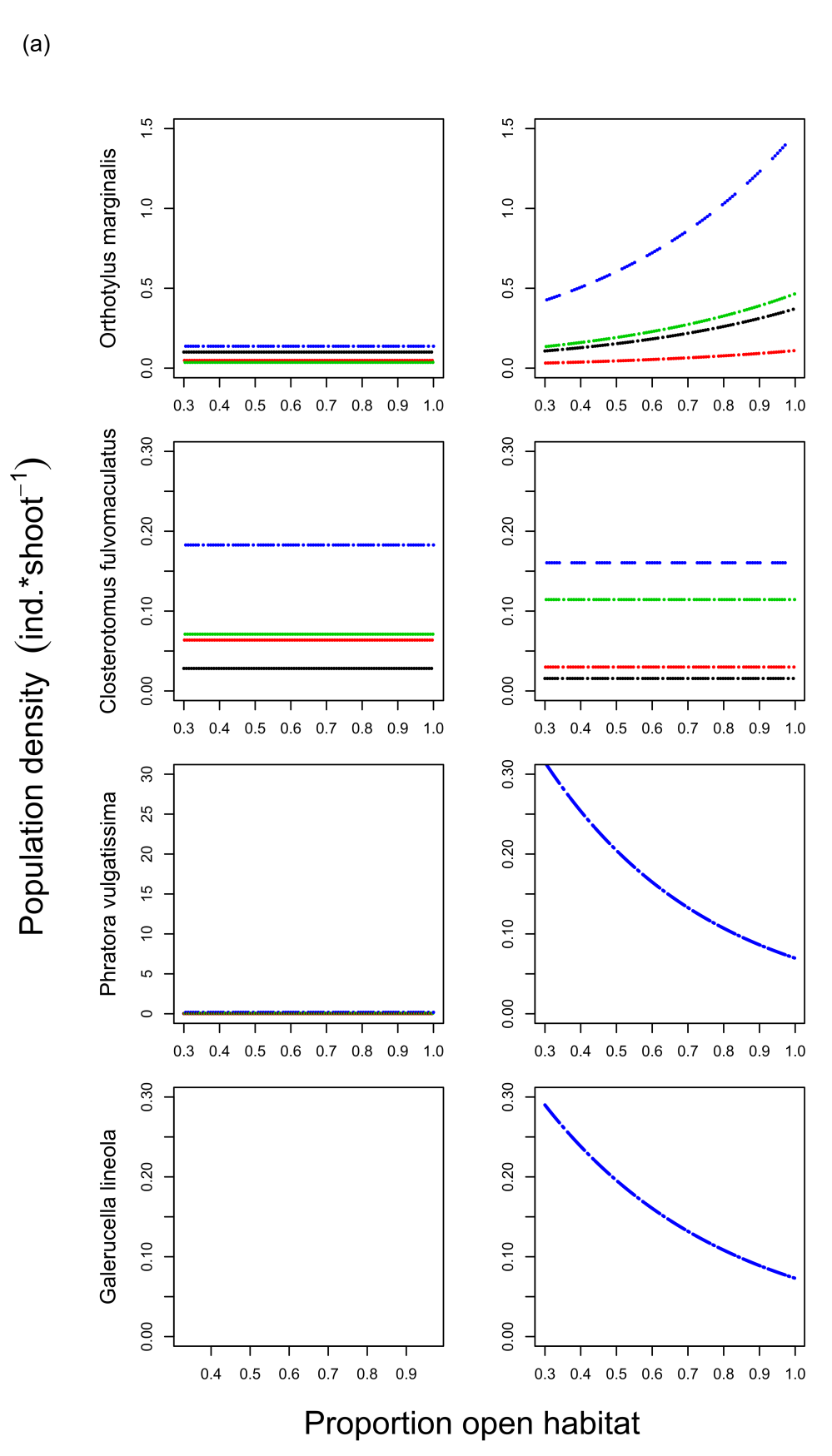

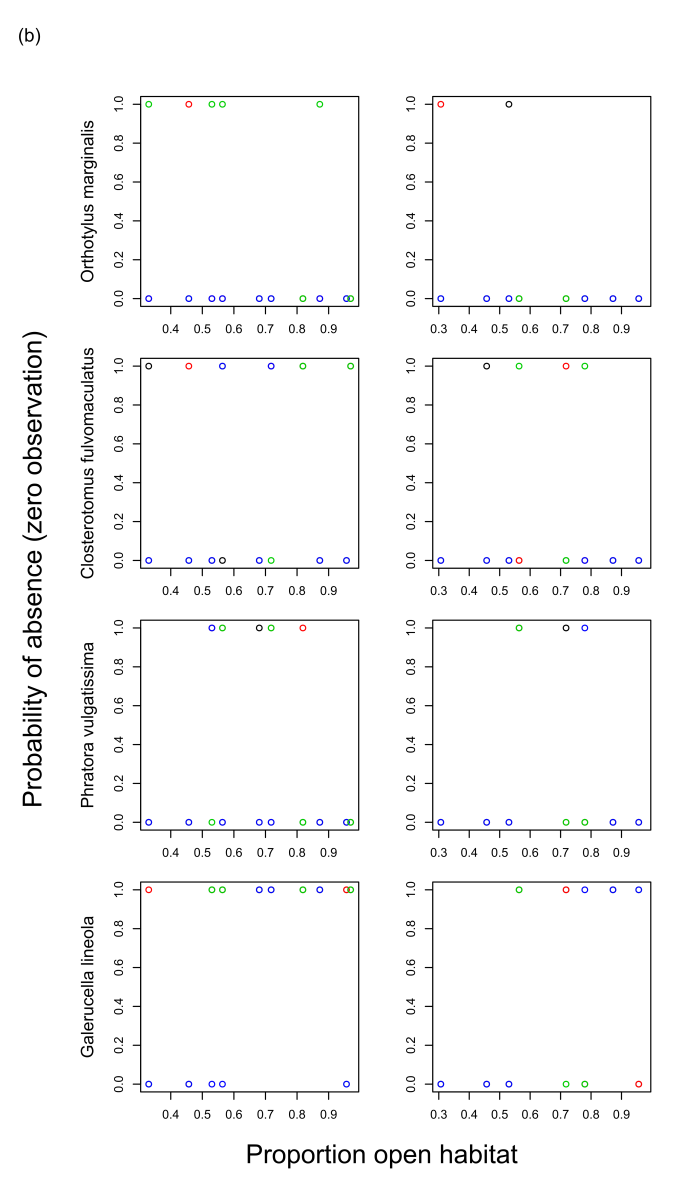
**

**Figure S2.** Alternative analyses with the aim to diagnose the impact of absence data (zero observations) on the relationship between population density (ind*shoot^-1^) of two mirid predators and two leaf beetle herbivores in SRC willow stands and the proportion of open habitat and/or year after harvest, two 4-year periods after coppice harvests. Predictions in the left panels are based on data from 2003–2006 and in the right panels on data from 2007–2010. (a) Analysis using a subset of the data, i.e. abundance ≥ 1 (Poisson distribution, log link) to describe population densities. Only variables with an overall significant effect on population density were used for the model predictions (single blue dotted line=no difference between years, four dotted lines=difference between observation years, red=year 1, black=year 2, green=year 3, red=year 4). Note that no results are presented for *G. lineola* in the first time period, due to the low number of observations (abundance ≥ 1). (b) Analysis all data using a binomial distribution (presence/absence) to describe the probability of absence, i.e. zero observations (binomial distribution, logit link). Open circles are raw data 0= abundance ≥ 1 and 1=zero observation, (red=year 1, black=year 2, green=year 3, red=year 4). Note that all data points cannot be seen since they are at the exact same position. No model predictions are shown since the open habitat variable was not significant in any of the models.

**Appendix S3**


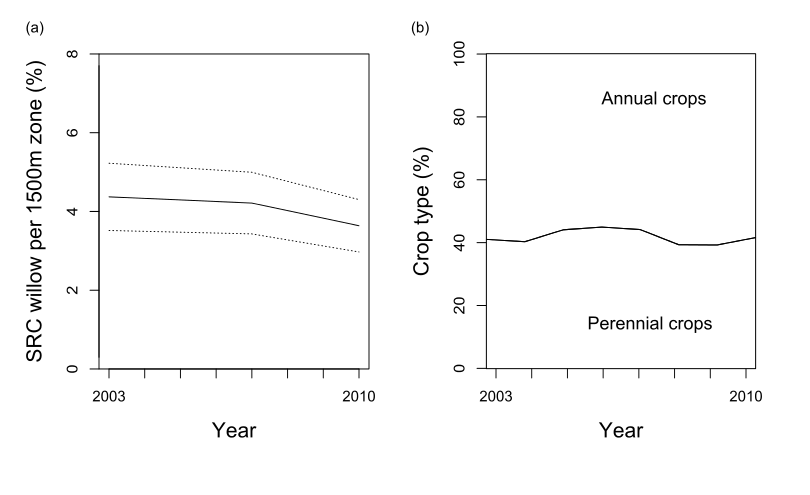


**Figure S3.** Land use changes over time (2003–2010) at landscape and regional scales. a) The percent SRC willow within 1500 m buffer zones from the patch edges (solid line = mean and dotted lines= standard error). b) The relative percentage of annual and perennial crop types (including SRC willow) comprising the total open land cover (~100 000 ha arable land) in the entire study area region.
